# Supplementary material for: Intention to leave the current health facility among healthcare workers in Ethiopia: Systematic review and meta-analysis
Source: PLOS Glob Public Health. 2024 Aug 14;4(8):e0003548. doi: 10.1371/journal.pgph.0003548 (PMC11324159; doi:10.1371/journal.pgph.0003548)
Supplement: S2 File — (DOCX) [file pgph.0003548.s002.docx]

A Table Showing Methodological Quality Assessment Results of Included Studies, 2023

| Study code | Was the sample frame appropriate to address the target population?(1) | Were study participants sampled in an appropriate way?(2) | Was the sample size adequate?(3) | Were the study subjects and the setting described in detail?(4) | Was the data analysis conducted with sufficient coverage of the identified sample?(5) | Were valid methods used for the identification of the condition?(6) | Was the condition measured in a standard, reliable way for all participants?(7) | Was there appropriate statistical analysis?(8) | Was the response rate adequate, and if not, was the low response rate managed appropriately?(9) | Total Yes/comment |
| --- | --- | --- | --- | --- | --- | --- | --- | --- | --- | --- |
| Adugna Endale Woldegiorgis, 2015 | No | Yes | No | Yes | Yes | Yes | Yes | Yes | Yes | 77.8% |
| Andualem Wubetie, 2020 | Ye | Yes | No | Yes | No | Yes | Yes | Yes | Yes | 66.7% |
| ASCHALEW MESFIN, 2022 | Yes | Yes | No | Yes | No | Yes | Yes | Yes | Yes | 77.8% |
| Aster Ferede, 2018 | Yes | Yes | Yes | Ye | Yes | Yes | Yes | Yes | Yes | 100% |
| Aynye Negesse Woldekiros, 2022 | Yes | Yes | Yes | Yes | Yes | Yes | Yes | Yes | Yes | 100% |
| Dawit Gebregziabher, 2020 | No | Yes | No | Yes | No | Yes | Yes | Yes | Yes | 66.7% |
| Endager Abera, 2014 | Yes | Yes | Yes | Yes | Yes | Yes | Yes | Yes | Yes | 100% |
| Endalkachew Dellie, 2019 | Yes | Yes | Yes | Yes | Yes | Yes | Yes | Yes | Yes | 100% |
| Fikirte Girma, 2021 | Yes | Yes | Yes | Yes | Yes | Yes | Yes | Yes | Yes | 100% |
| Girma Alem Getie, 2015 | Yes | Yes | Yes | Yes | Yes | Yes | Yes | Yes | No | 88.9% |
| Hailay Abrha Gesesew, 2016 | Yes | Yes | Yes | Yes | Yes | Yes | Yes | Yes | No | 88.9% |
| Hangasu Udess, 2020 | Yes | Yes | No | Yes | No | Yes | Yes | Yes | Yes | 77.8% |
| Nigusu Worku, 2019 | Yes | Yes | Yes | Yes | Yes | Yes | Yes | Yes | Yes | 100% |
| Tilahun Mekonnen, 2022 | Yes | Yes | Yes | Yes | Yes | Yes | Yes | Yes | Yes | 100% |
