# Supplementary material for: Intention to leave the current health facility among healthcare workers in Ethiopia: Systematic review and meta-analysis
Source: PLOS Glob Public Health. 2024 Aug 14;4(8):e0003548. doi: 10.1371/journal.pgph.0003548 (PMC11324159; doi:10.1371/journal.pgph.0003548)
Supplement: Attachment — Submitted filename: PGPH-D-23-02236_reviewer.pdf [file pgph.0003548.s004.pdf]

PLOS Global Public Health

Intention to Leave the Current Health Facility among Healthcare Workers in Ethiopia: Systematic Review and Meta-analysis

--Manuscript Draft--

Manuscript Number:	PGPH-D-23-02236
Article Type:	Research Article
Full Title:	Intention to Leave the Current Health Facility among Healthcare Workers in Ethiopia: Systematic Review and Meta-analysis
Short Title:	Intention to Leave the Current Health Facility among Healthcare Workers
Corresponding Author:	Gizew Dessie Asres, B.Pharm, MPH<br>Amhara Regional Health Bureau: Amhara National Regional Health Bureau<br>Bahir Dar, Amhara ETHIOPIA
Order of Authors:	Gizew Dessie Asres, B.Pharm, MPH
	Yeshiwork Kebede Gessesse, MSc
Keywords:	Current Health Facility; Ethiopia; Healthcare Workers; Intention to Leave; Systematic Review and Meta-analysis
Abstract:	Background: Strengthening workforce management to address retention challenges is a worldwide concern. Ethiopia did different human resource reforms to improve retention and performance of available health workforce to step up towards universal health coverage. However, there is limited and fragmented research on intention to leave their current facility and related factors on healthcare workers. This study was done to assess the Ethiopian pooled national prevalence of intention to leave and related factors from 2013 to 2022.<br>Methods: This systematic review and meta-analysis followed PRISMA guidelines. The authors prepared a review protocol per JBI manual for evidence synthesis and got registered from PROSPERO for transparency. The protocol details criteria the reviewers have used to include and exclude studies.<br>Results: Pooled proportion of intention to leave among health workers in Ethiopian healthcare setting was found to be 63.317% (95% CI (58.606 - 67.904)) for Random effect model at Q= Q=141.5689 (I2(inconsistency)= 90.82%, P < 0.0001). Of assessed factors only organizational policy OR = 0.29 (0.14-0.61) was found to be a significantly associated factor for health workers' intention to leave at healthcare setting in Ethiopia.<br>Conclusion: More than 6 in 10 of the healthcare workers in Ethiopia were ready to leave their current health facility. This result was higher than studies done in other parts of the world, even in African countries. The associated factor for health workers' intention to leave their current health facility was only organizational policy and health authorities should improve their organizational policies in line with their employees' concerns.<br>Keywords: Current Health Facility; Ethiopia; Healthcare Workers; Intention to Leave; Systematic Review and Meta-analysis
Additional Information:	
Question	Response
Financial Disclosure<br><br>Enter a financial disclosure statement that describes the sources of funding for the work included in this submission and the role the funder(s) played. This includes grants and any commercial funding of the work or authors.	No funding was received for this project.

Powered by Editorial Manager® and ProduXion Manager® from Aries Systems Corporation

This statement will be typeset if the manuscript is accepted for publication.<br><br>*Please review the submission guidelines and the instructions link below for detailed requirements and guidance.*	
**Competing Interests**<br><br>On behalf of all authors, disclose any competing interests that could be perceived to bias this work.<br><br>This statement will be typeset if the manuscript is accepted for publication.<br><br>*Please review the instructions link below and PLOS Global Public Health's competing interests policy to determine what information must be disclosed at submission.*	The authors have no conflict of interest.
**Data Availability**<br><br>Before publication, Authors are required to make fully available and without restriction all data underlying their findings. Please see our PLOS Data Policy page for detailed information on this policy.<br><br>A **Data Availability Statement**, detailing where the data can be accessed, is required at first submission. Insert your Data Availability Statement in the box below.<br><br>Please see the data reporting section of our submission guidelines for instructions on what you need to include in your Data Availability Statement.	All necessary information was attached to this manuscript. Whenever an additional data set is needed, Data sharing is applicable upon reasonable request to the corresponding author.

This statement will be typeset if the manuscript is accepted for publication.<br><br>*PLOS allows rare exemptions to address legal and ethical concerns. If you have legal or ethical restrictions, please use the box below to detail these in full sentences for the Journal team to consider.*	

Powered by Editorial Manager® and ProduXion Manager® from Aries Systems Corporation

Manuscript

Click here to access/download;Manuscript;Manuscript Intention to leave.docx

Intention to Leave the Current Health Facility among Healthcare Workers in Ethiopia: Systematic Review and Meta-analysis

Gizew Dessie Asres [1, *], Yeshiwork Kebede Gessesse[2]

1. Hospital Performance Monitoring and Evaluation Team Leader, Asrade Zewude Memorial Primary Hospital, Bure City Administration, Amhara Regional State, Ethiopia.

   *Corresponding Author

   E-mail: gizew.dessie@gmail.com

   Tel: +251910183763

   ORCID: https://orcid.org/0000-0002-3979-3998

2. Integrated Emergency Surgical Officer (IESO) (Ob-Gyn and General Surgery), Durbete Primary Hospital, Amhara, Ethiopia.

   Email: yeshiworkk18@gmail.com

   Tel: +251920759628

   ORCID: https://orcid.org/0000-0002-5425-2241

Abstract

Background: Strengthening workforce management to address retention challenges is a worldwide concern. Ethiopia did different human resource reforms to improve retention and performance of available health workforce to step up towards universal health coverage. However, there is limited and fragmented research on intention to leave the current facility and related factors on healthcare workers. This study was done to assess the Ethiopian pooled national prevalence of intention to leave and related factors from 2013 to 2022.

Methods: This systematic review and meta-analysis followed PRISMA guidelines. The authors prepared a review protocol per JBI manual for evidence synthesis and got registered from PROSPERO for transparency. The protocol details criteria the reviewers have used to include and exclude studies.

Results: Pooled proportion of intention to leave among health workers in Ethiopian healthcare setting was found to be 63.317% (95% CI (58.606 - 67.904)) for Random effect model at Q= Q=141.5689 (I^2(inconsistency)= 90.82%, $P < 0.0001$). Of assessed factors only organizational policy OR = 0.29 (0.14-0.61) was found to be a significantly associated factor for health workers' intention to leave at healthcare setting in Ethiopia.

Conclusion: More than 6 in 10 of the healthcare workers in Ethiopia were ready to leave their current health facility. This result was higher than studies done in other parts of the world, even in African countries. The associated factor for health workers' intention to leave their current health facility was only organizational policy and health authorities should improve their organizational policies in line with their employees' concerns.

Keywords: Current Health Facility; Ethiopia; Healthcare Workers; Intention to Leave; Systematic Review and Meta-analysis

Introduction

Health workforce shortage in healthcare settings is a global challenge of the healthcare system, further exacerbated by the COVID-19 pandemic bourdon from 2019 onwards(1). Among contributing factors to health workforce shortage at the facility level is a turnover of experienced health workers plays a vital role. Turnover intention and intention to leave are used interchangeably in this project. The actual level of health workforce turnover at a health facility can easily be calculated from annual exit data retrospectively. However, a prospective and a proxy indicator to predict turnover is by measuring turnover intention, more amenable to intervention based on the assessment result of each health facility. Turnover intention or intention to leave is employees' willingness to leave their organisation within a definite period (2–4)

Turnover seems simply to leave employees at their facility or transfer to other health facilities and replace their vacant posts with new employees having equivalent competency. In the case of the health workforce, there is still a supply shortage of some disciplines to replace the vacant post and this creates a shortage at one health facility and may create excess at other health facilities. High turnover of employees at health facilities impairs workflow (5), leads to lower quality of care (6), and incurs considerable financial costs (7). Retaining experienced health workers in health facilities is a challenge in many countries worldwide (8).

The sustainability of an organization depends on its resources, specifically the human resource (9). Experienced human resources help organizations to have a competitive advantage(10). Turnover, the voluntary separation of experienced human resources, highly affects the health facilities' service quality (11). Intention to leave the current facility is a proxy indicator of actual turnover (12,13) So far researchers identified domains that influence health workforce turnover as: leadership and management, education and career advancement, staffing level, professional issues, support at work, personal influences, demographic influences, and financial remuneration(14).

Studies showed a 15-27% annual turnover rate of nurses in Denmark, the US, Canada, and Australia (15–18). A systematic review in China revealed that 30.4% of primary healthcare workers and 47% of general practitioners had the intention to leave their working facility. The factors identified in this study were categorized as demographic factors, job characteristic factors, and job satisfaction factors (19,20). The prevalence of intention to leave among psychiatry nurses was 44% in a study done in Japanese hospitals (21). 61.9% of pharmacists had the intention to be in Riyadh, Saudi Arabia (22).

Low- and middle-income countries should strengthen the institutional environment for health workforce production deployment, retention and performance management(8). The prevalence of intention to leave among nurses in sub-Saharan Africa was 50.74 % (23). Another intention to

leave study among health workers showed that the proportion of intention to leave was 18.8%, 26.5%, and 41.4% in Tanzania, Malawi, and South Africa respectively (24).

The Ethiopian Ministry of Health assessed and redesigned various financial and non-financial incentives for the health workforce's motivation and retention such as standardized duty allowance (overtime payments) for all health professionals, risk allowance for selected health cadres with relatively highest risks of exposure to health hazards, house allowance or providing free residential houses for physicians. However, the implementation of these initiatives (proposed and approved by Mo[illegible]s not uniform/consistent in various regional states. Besides, the health workers perceived the packages were neither adequate nor inclusive of all health cadres(25).

In Ethiopia, there is no representative and summarized data on health professionals' intention to leave their current health facility. Some studies on intention to leave did not clearly state whether it is the intention to leave their current profession or their current working facility. The paucity of previous studies showed inconsistent prevalence that ranges from 30.4 % to 80.6% (26,27). Therefore, this systematic review and meta-analysis aimed to estimate the pooled prevalence of health professionals' intention to leave their current health facility and factors associated with it in Ethiopia using previous studies done from 2013-2022.

Methods

A systematic review and meta-analysis following PRISMA guidelines was done to assess the level of intention to leave and factors related to it among healthcare workers in healthcare settings in Ethiopia (28). A review protocol was developed as per the JBI manual for evidence synthesis to pre-define the objectives and methods of the systematic review (29). The protocol details the criteria the reviewers have used to include and exclude studies. This protocol was registered at the International Prospective Register of Systematic Reviews (PROSPERO) ID=CRD42022312559: https://www.crd.york.ac.uk/prospero/display_record.php.

Review Question

This systematic review was guided by the following review questions based on the condition, context and population (CoCoPop) framework;

1. What is the pooled prevalence of intention to leave their current health facility among healthcare workers in Ethiopia?
2. What are the factors for the intention to leave the current health facility among healthcare workers in Ethiopia?
3. Is there a variation of intention to leave among healthcare categories in Ethiopia?

Search strategy

We conducted a comprehensive search of PubMed, Scopus, Web of Science, Cochrane, Google Scholar and African Index Medicus databases and grey literature like the WHO library from 8 June 2022 to 30 May 2023. The search terms used at PubMed were 'Health worker' OR 'health staff' OR 'health care staff' OR 'human resource for health' OR 'health employee' OR 'health professional' AND 'health care setting' OR health facility OR health institution OR hospital OR health centre OR clinic OR health post AND determinants OR factors OR predictors AND intention to leave OR turnover intention OR intention to quit AND Ethiopia.

Inclusion criteria

The inclusion criteria were based on CoCoPop mnemonic (Condition, Context, and Population) and used for reviewers to screen and select articles for prevalence data on intention to leave and related factors (**Table** 1).

Table 1 Inclusion and exclusion criteria of studies, 2023

Included studies	Excluded studies
1. Studies were done on health workers' intention to leave their current facility between 2013 and 2022 in Ethiopia<br>2. Observational studies with analytical parts.<br>3. Intention to leave Studies done at all levels of health care settings or facilities.<br>4. Intention to leave studies on all categories of health workers (clinical and other service providers).<br>5. Intention to leave studies done on private or public healthcare settings<br>6. Studies written in the English language.<br>**7.** Methodological quality >50%.	1. Health workers of the Woreda Health Office, Zonal Health Department, Regional Health Bureau, Ministry of Health, blood bank, Ethiopian Food Drug Administration, professional associations and health science colleges<br>2. Intention to leave studies done on health workers in health care settings outside of Ethiopia<br>3. Intention to leave studies done on health workers in health care settings before 2013<br>4. Studies investigated the Intention to leave (change) current job.

Study Screening and Selection

The process of screening was conducted in two levels. Level one was based on the titles and abstracts of the paper. In the second level, papers that have passed the screening level were downloaded and evaluated against the inclusion criteria. All selected articles from this stage were saved in a separate folder for quality assessment and those that passed the quality assessment were used for this evidence synthesis. Papers that were excluded based on the full-text assessment against the inclusion and exclusion criteria were justified. The entire screening and selection process was undertaken using Rayyan software(30) independently by two authors, who

later compared their results from the software and there were no major discrepancies and minor discrepancies were resolved based on consensus between the two authors.

Quality Assessment (Appraisal) of Studies

A critical appraisal tool based on the study design was used to assess the methodological quality assessment of included studies. A critical appraisal checklist developed by JBI for prevalence studies has been used for this study (**Annex 1**). Two reviewers GDA and YKG have appraised the studies independently using the abovementioned tool. The tool encompassed 9 criteria for rating different quality elements and studies scored 5 and above out of 9 were included in this meta-analysis study. During the quality assessment disagreements were solved through discussion and before an agreement reached weighted kappa index was done, which was 98.33% (29,31).

Data Extraction

JBI Standardized data extraction template was used to ensure the extraction of the same types of data across the included studies. The two reviewers GDA and YKG extracted the entire necessary data independently using the SRDR+ online platform (32). The data collection included the following items; Study details, Study Method and Results.

Data synthesis and Meta-analysis

Data extracted from relevant studies were analyzed using tables, graphs, and other diagrams to investigate how studies compare to each other. Meta-analysis was conducted to pool health workers level of intention to leave by using random and fixed effects models. The data from all included studies were synthesized according to outcome variables and study designs using Meta Essentials v 1.5 (33,34) and MedCalc v 20.114 (35) statistical tools. The extent of heterogeneity across studies was checked using the Q-test and I^2-test (I squared >50% indicating significant heterogeneity). To study the effect of covariates on the pooled effect size and the heterogeneity across studies, subgroup analysis was conducted on; the sample size of individual studies, year of publication, health care setting, region and health worker categories. The effect of covariates on the pooled effect size was considered significant when the p-value was <0.05 or their 95% CI did not cross zero.

Results

Screening and selection process of included studies

During the bibliographic data base search, a total of 2, 972 studies were identified. Of which 1, 005 were duplicates and removed. Of 1, 967 remaining studies, 1, 903 were found to be unrelated and excluded based on title and abstract screening. Finally,64 full text articles were downloaded for further evaluation against eligibility criteria and 15 full text articles excluded as

they missed prevalence of turnover intention data and 31 full text articles found unrelated for this systematic review and excluded. Four redundant full text articles were excluded. At last, we ended up with 14 full text articles for evidence synthesis (**Fig 1**).

Characteristics of included studies

Among 14 included studies published from 2013-2022 in Ethiopia, the smallest sample size was 102 on a study done in Addis Ababa and the largest one was 568 on a study done in Amhara regional state. The study settings included were health centers and hospitals. These studies involved a total of 4575 participants (**Table 2**).

Methodological quality of included studies

Methodological quality assessment was done using the JBI's Prevalence Studies Critical Appraisal Checklist. Assessment was done by two independent assessors, GDA and YKG. Discrepancies between assessors were resolved through consensus and mutual understanding. The assessment results revealed that quality of included studies range from 66.7% to 100% (**Annex 2)**.

Findings of the Review

Based on 14 included studies with 4575 participants, the pooled intention to leave their current health facilities among health workers at Ethiopian health care setting was found to be 63.25% ((95% CI (61.84 - 64.65)) for fixed effect model and 63.32% (95% CI (58.606 - 67.904)) for Random effect model at Q=141.57 (I^2(inconsistency)= 90.82%, P < 0.0001). Only the results from random effect model were used for further discussion as there was significant heterogeneity between studies (**Table 3**).

Table 3 Level of health worker Intention to leave at health care settings in Ethiopia (n=14), 2023

Study ID	Sample size	Proportion (%)	95% CI	Weight (%)	
				Fixed	Random
Adugna Endale Woldegiorgis, 2015	252	48.413	42.095 to 54.768	5.51	7.06
Andualem Wubetie, 2020	102	77.451	68.111 to 85.137	2.24	6.05
ASCHALEW MESFIN, 2022	242	57.851	51.359 to 64.149	5.30	7.03
Aster Ferede, 2018	568	61.268	57.123 to 65.294	12.40	7.54
Aynye Negesse Woldekiros, 2022	392	80.612	76.346 to 84.409	8.56	7.36
Dawit Gebregziabher, 2020	148	64.865	56.598 to 72.524	3.25	6.54
Endager Abera, 2014	394	52.538	47.477 to 57.560	8.61	7.37
Endalkachew Dellie, 2019	336	65.476	60.124 to 70.552	7.34	7.27
Fikirte Girma, 2021	402	70.647	65.930 to 75.056	8.78	7.38
Girma Alem Getie, 2015	372	59.409	54.225 to 64.441	8.13	7.33
Hailay Abrha Gesesew, 2016	367	59.401	54.181 to 64.467	8.02	7.33
Hangasu Udess, 2020	256	54.687	48.370 to 60.895	5.60	7.07
Nigusu Worku, 2019	382	67.801	62.860 to 72.464	8.35	7.35
Tilahun Mekonnen, 2022	362	64.917	59.756 to 69.832	7.91	7.32
Total (fixed effects)	4575	63.249	61.835 to 64.646	100.00	100.00
Total (random effects)	4575	63.317	58.606 to 67.904	100.00	100.00

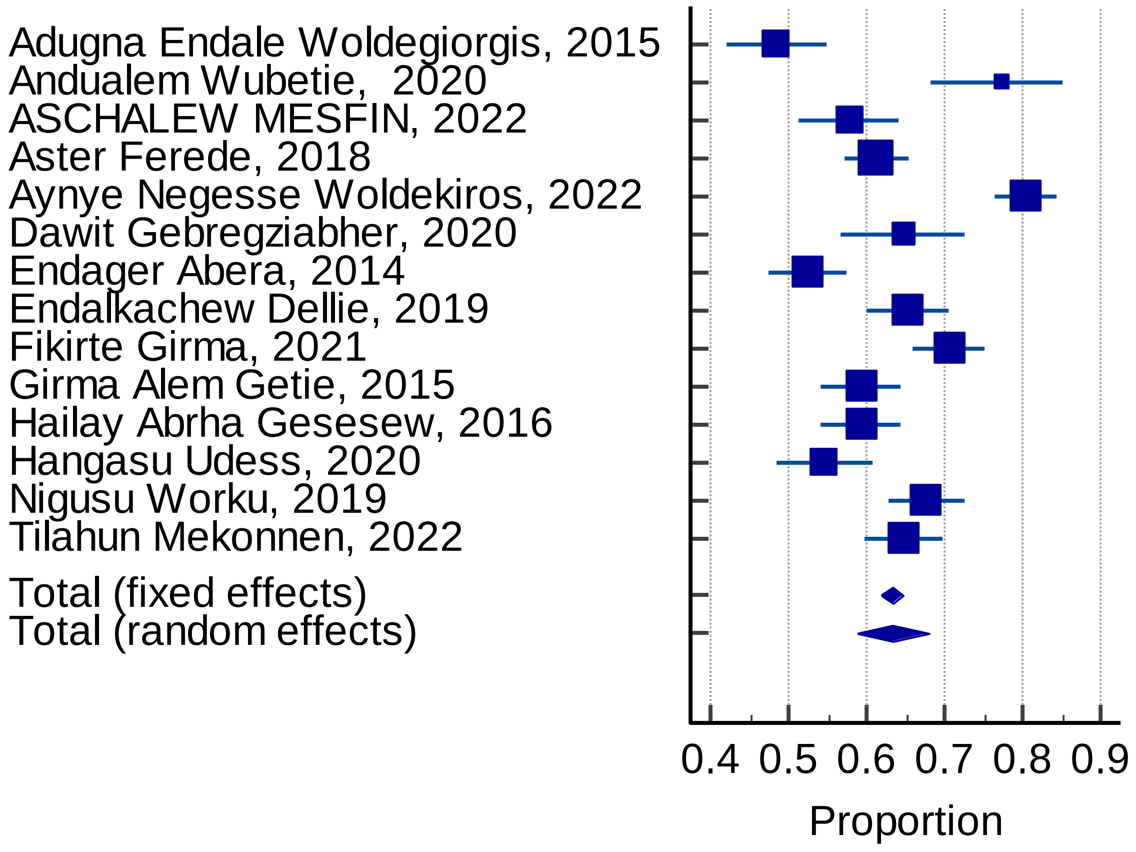

Fig 2 Forest plot showing effect size of intention to leave the current health facility among health workers in Ethiopia, 2023

Publication bias analysis of included studies

The Egger's test and symmetric funnel plot showed that studies included in this meta-analysis have no publication bias (**Table 4 and Fig 3**).

Table 4 Egger's and Begg's test for publication bias

**Egger's test**	
Intercept	0.02467
95% CI	-10.6577 to 10.7071
Significance level	P = 0.9961

Begg's test	
Kendall's Tau	-0.1209
Significance level	P = 0.5470

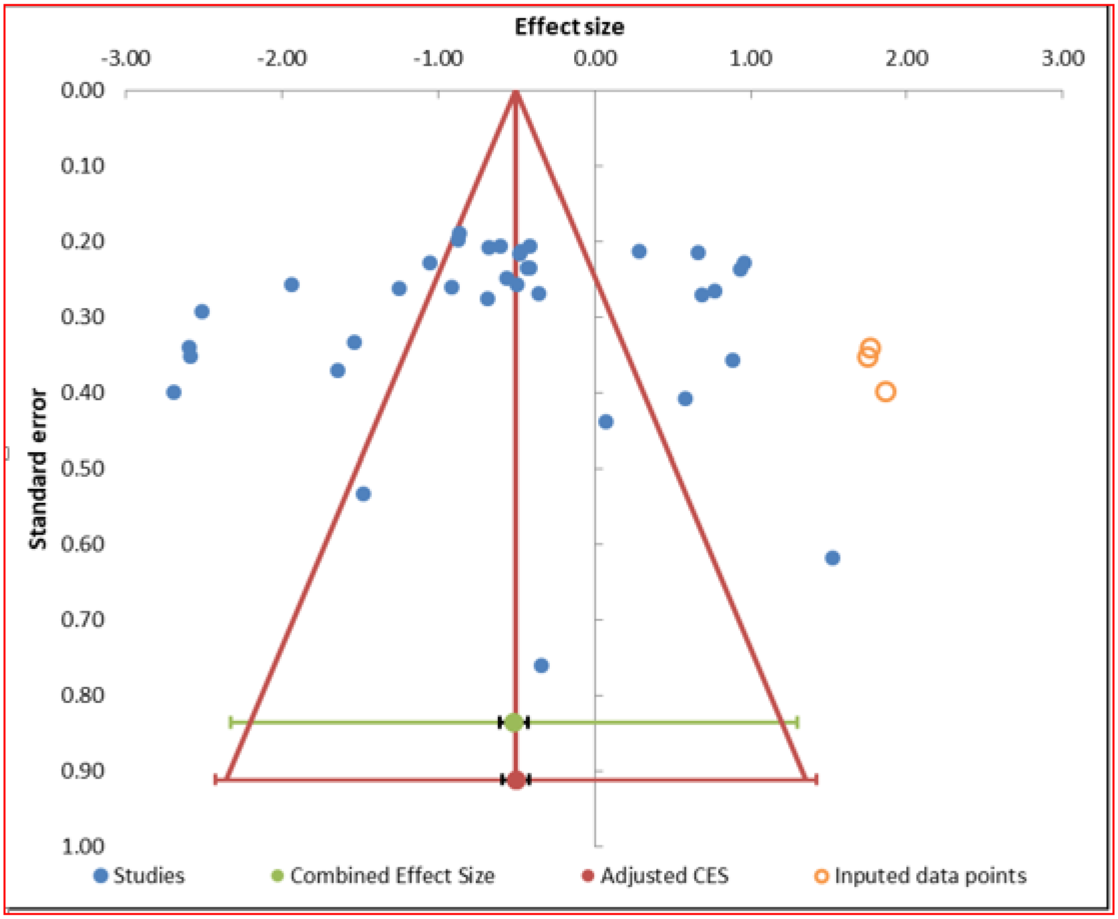

Fig 3 Funnel plot showing symmetry of included articles

Subgroup Analysis of Included Studies

To investigate source of heterogeneity, sub group analysis has been done on sample size, study area, participant's profession, year of publication and health facility type. All the above listed variables were significant source of statistical heterogeneity of which year of publication was the highest source of heterogeneity (I^2=99.02%, **Table 5**).

Table 5 Subgroup analysis of included studies for systematic review and meta-analysis on intention to leave their current health facility, 2023

**Subgroup Name**		**Odds Ratio**	**95% CI**	**Weight**
**Location** Q=113.48, T^2 =0.22, PQ=0.001, I^2=96.48%, 95%CL Pooled Odds Ratio=0.97 (0.52, 1.81), Weighted Average=12.80%	Addis Ababa	2.09	1.77, 2.47	20.45%
	Amhara	0.82	0.73, 0.93	20.76%
	Gambela	0.53	0.41, 0.68	19.62%
	Oromia	0.86	0.74, 0.99	20.61%
	Tigray	1.08	0.77, 1.52	18.56%
**Participants** Q=58.55, T^2 =0.20, PQ=0.001, I^2=96.58%, 95%CL Pooled Odds Ratio=1.05(0.39, 2.82), Weighted Average=12.32%	All	0.69	0.60, 0.79	33.88%
	Lab	1.12	0.89, 1.41	32.42%
	Nurse	1.51	1.30, 1.75	33.70%
**Publication Year** Q=101.93, T^2 =0.39, PQ=0.001, I^2=99.02%, 95%CL Pooled Odds Ratio=1.00(0.00, 273.88), Weighted Average=3.33%	2014-2018	0.64	0.57, 0.73	50.00%
	2019-2022	1.56	1.38, 1.76	50.00%
**Response Rate** Q=10.80, T^2 =0.04, PQ=0.001, I^2=90.74%, 95%CL Pooled Odds Ratio=1.00 (0.16, 6.19), Weighted Average=31.53%	<95	0.87	0.77, 0.98	50.00%
	>=95	1.15	1.02, 1.30	50.00%
**Sample Size** Q= 55.41, T^2 =0.11, PQ=0.001, I^2=92.78%, 95%CL Pooled Odds Ratio=1.05 (0.68, 1.61), Weighted Average=27.04%	100-200	1.39	1.05, 1.84	18.42%
	201-300	0.62	0.53, 0.73	20.64%
	301-400	1.14	1.01, 1.28	21.15%
	401-500	1.45	1.16, 1.82	19.51%
	501-600	0.92	0.77, 1.10	20.28%
**Study Setting** Q=25.92, T^2 =0.10, PQ=0.001, I^2=96.14%, 95%CL Pooled Odds Ratio=1.01 (0.06, 17.14), Weighted Average=12.99%	Hospital	1.25	1.11, 1.41	50.00%
	Hosp and HC	0.80	0.71, 0.90	50.00%

Factors Associated with Health Worker's Intention to Leave at Health Care Settings in Ethiopia

Fourteen included studies used for pooled prevalence of intention to leave and analysis of factors associated with it. Factors assessed were; autonomy, co- worker relationship, job satisfaction, organizational policy, payment and benefits system, performance appraisal, recognition, training and development opportunity, and workload. Of which only organizational policy OR = 0.29(0.14-0.61) was found to be significantly associated factors for intention to leave of health workers at health care setting in Ethiopia (**Table 6**).

Discussion

The aim of this systematic review and meta-analysis was to assess the related factors and pooled prevalence of intention to leave their current health facilities among health workers in Ethiopia. We studied intention to leave as a direct predictor and proactive approach to determine the pooled prevalence of turnover among health workers in Ethiopia.

The distribution of included 14 studies throughout Ethiopia was 21.4%, 42.9%, 7.1%, 21.4% and 7.1% in Addis Ababa, Amhara, Gambela, Oromia and Tigray respectively. The smallest sample size among included studies was 102 and the largest one was 568 from studies done in Addis Ababa and Amhara respectively. The response rate of studies ranges from 87% to 100% and publication year was from 2015 to 2022.

From this review, we synthesized that among 14 individual studies, the pooled intention to leave their current health facility among health workers in Ethiopia was 63.32% (95% CI (58.61 - 67.90)) for Random effect model at Q=141.57 (I^2 (inconsistency) = 90.82%, P < 0.0001). We used only the results from the random effect model as there was significant heterogeneity between studies. The highest prevalence of intention to leave was 80.6% and the lowest was 48.4% as reported by a study done in Addis Ababa and Gambela, in 2022 and 2015 respectively.

This finding was higher than a study done on general practitioners in 2020 globally which was reported as 47% (20). The difference may be due to variations in characteristics of study participants, socio economic status countries and variations in health work force retention mechanisms.

Our finding is higher than a cross sectional study of New Jersey hospital nurses intention to leave their hospital (36.5%) (36). This difference may be due to variations of study design and study participants, all health workers versus nurses. When compared with a report of 30.4% intention to leave Chinese primary health workers by a systematic review and meta-analysis done in 2020, our finding was by far higher(37). The difference may be due to variations in characteristics of study participants, socio economic status countries and variations in health work force retention mechanisms.

This finding is higher when compared to African countries like Tanzania(18.8%), Malawi (26.5%) and South Africa (41.4%)(24). This difference may be due to variations with respect to study procedures, participants and study settings. Significant heterogeneity was observed in regional states on intention to leave as 64.86% in Tigray regional state, 60.81% in Amhara regional state, 75.78% in Addis Ababa city administration and 60.20% in Oromia regional state. Prevalence of intention to leave Ethiopia at health care settings was 65.98% and 60.80% at hospitals only and, studies done at both hospitals and health centers levels respectively. Based on health worker categories, the intention to leave was 60.56%, 65.48% and 70.22% among studies that include all health categories, studies of laboratories only and studies of nurses only respectively.

The association between Intention to leave the current health facility and associated factors have been tested. The factors tested are; autonomy, co-worker relationship, job satisfaction, organizational policy, payment and benefits system, performance appraisal, recognition, training and development opportunity and workload. However only organizational policy was found to have a significant association with intention to leave their current health facility as pooled odds ratio 0.29 at 95% CL (0.14-0.61). The odds of intention to leave among health care staff who believe their organizational policy is conducive was 0.71 times less than that of health care staff who believe their organizational policy was not conducive.

Limitation

This systematic review and meta-analysis have some limitations. First, the included studies were heterogeneous with respect to study procedures, participants and study settings. Accordingly, our findings were summarized on a broader level, which inherently suppresses some of the unique features of different approaches. Second, the majority of studies were based on a cross-sectional study design that limits the precision of intention to leave results. Third, the included articles were only full-text articles freely accessible which compromises the quality and generalizability of the review.

Conclusion

From this review, we synthesized that the proportion of pooled intention to leave healthcare settings among healthcare workers in Ethiopia was more than 6 in 10 health workers. This result was higher than studies done in other parts of the world, even the African countries. This discrepancy might be due to variations in socio-economic, political and studied health worker categories. Based on this systematic review and meta-analysis, the associated factor for health workers' intention to leave their current health facility was only organizational policy.

Recommendation

Health facilities in Ethiopia should strive to have shared, transparent and conducive organizational policies to retain health workers. To be more specific and precise, the authors

recommend further systematic review and meta-analysis on a departmental basis and conduct an original study on administrative staff who are working in finance, human resources and health professionals who are small in number at health facilities, like environmental health.

Declarations

Ethics approval and consent to participate

NA

Consent for publication

NA

Availability of data and materials

All necessary information was attached to this manuscript. Whenever an additional data set is needed, Data sharing is applicable upon reasonable request to the corresponding author.

Competing interests

The authors have no conflict of interest.

Funding

No funding was received for this project.

Authors' contributions

GDA and YKG contributed to the conception and design of the review. GDA and YKG were responsible for screening, data extraction, synthesis, and analysis of the data. GDA drafted the manuscript, which was critically revised by YKG. Rechecking together was done before the final stage of the manuscript.

Acknowledgements

It is a great pleasure to thank Yirga Yeshiwas Alefe, CEO of Bure Asrade Zewude Hospital for frequent consultation on how to conduct a systematic review. The authors would like to acknowledge the contributions made by MedCalc for 14-day free trial meta-analysis database and freely accessed Meta essentials database for meta-analysis. We would also like to acknowledge JBI for the live webinar by Associate Professor Edoardo Aromataris on 'How to conduct systematic review: Latest guidance' and Campbell collaborative on how to calculate effect size for meta-analysis by Prof. David Wilson.

Abbreviations

CoCoPop- condition, context and population framework; HEW-Health Extension Worker; JBI-Joanna Briggs Institute; PRISMA- Preferred Reporting Items for Systematic Reviews and Meta-Analyses; PROSPERO-International Prospective Register of Systematic Reviews; SRDR+-Systematic Review Data Repository plus; WHO-World health organization

Authors' information

[1]Hospital Performance Monitoring and Evaluation Team Leader, Asrade Zewude Memorial Primary Hospital, Bure City Administration, Amhara Region, Ethiopia. [2]Licensed consultant on health and health-related issues, Gizew Pharmacy, Bure City Administration, Amhara region, Ethiopia. [3]Integrated Emergency Surgical Officer (IESO) (Ob-Gyn and General Surgery), Durbete Primary Hospital, Amhara, Ethiopia.

References

1. Poon YSR, Lin YP, Griffiths P, Yong KK, Seah B, Liaw SY. A global overview of healthcare workers' turnover intention amid COVID-19 pandemic: a systematic review with future directions. Hum Resour Health. 2022;20(1):1–18.

2. Lazzari M, Alvarez JM, Ruggieri S. Predicting and explaining employee turnover intention. Int J Data Sci Anal. 2022 Sep 1;14(3):279–92.

3. Tanova C, Holtom BC. Using job embeddedness factors to explain voluntary turnover in four European countries. Int J Hum Resour Manag. 2008;19(9):1553–68.

4. Ait Alla A, RAJÂA O. A Review of the Literature on Employee Turnover. Am Int J Soc Sci. 2019;8(3).

5. Hayes LJ, O'Brien-Pallas L, Duffield C, Shamian J, Buchan J, Hughes F, et al. Nurse turnover: a literature review–an update. Int J Nurs Stud. 2012;49(7):887–905.

6. Halter M, Boiko O, Pelone F, Beighton C, Harris R, Gale J, et al. The determinants and consequences of adult nursing staff turnover: a systematic review of systematic reviews. BMC Health Serv Res. 2017 Dec 15;17(1).

7. Li YIN, Jones CB. A literature review of nursing turnover costs. J Nurs Manag. 2013;21(3):405–18.

8. Organization WH. Global strategy on human resources for health: Workforce 2030. Who. 2016. 64 p.

9. Martin A, Roodt G. Perceptions of organisational commitment, job satisfaction and turnover intentions in a post-merger South African tertiary institution. SA J Ind Psychol. 2008;34(1):23–31.

10. Chegini. The Relationship between Organizational Justice and Organizational Citizenship Behavior. Am J Econ Bus Adm. 2009;1(2):173–6.

11. Kalifa T, Ololo S, Tafese F. Intention to Leave and Associated Factors among Health Professionals in Jimma Zone Public Health Centers, Southwest Ethiopia. Open J Prev Med. 2016;06(01):31–41.

12. Namin BH, Øgaard T, Røislien J. Workplace incivility and turnover intention in organizations: A meta-analytic review. Vol. 19, International Journal of Environmental Research and Public Health. 2022.

13. Hou H, Pei Y, Yang Y, Lu L, Yan W, Gao X, et al. Factors Associated with Turnover Intention Among Healthcare Workers During the Coronavirus Disease 2019 (COVID-19) Pandemic in China. Risk Manag Healthc Policy. 2021;14:4953–65.

14. Marufu TC, Collins A, Vargas L, Gillespie L, Almghairbi D. Factors influencing retention among hospital nurses: Systematic review. Vol. 30, British Journal of Nursing. MA Healthcare London; 2021. p. 302–8.

15. Duffield CM, Roche MA, Homer C, Buchan J, Dimitrelis S. A comparative review of nurse turnover rates and costs across countries. Vol. 70, Journal of Advanced Nursing. John Wiley & Sons, Ltd; 2014. p. 2703–12.

16. Mathisen J, Nguyen TL, Jensen JH, Rugulies R, Rod NH. Reducing employee turnover in hospitals: Estimating the effects of hypothetical improvements in the psychosocial work environment. Scand J Work Environ Health. 2021 Sep 9;47(6):456–65.

17. 2020 NSI National Health Care Retention & RN Staffing Report - Published by: NSI Nursing Solutions, Inc. www.nsinursingsolutions.com. [Internet]. [cited 2023 Jul 26]. Available from: https://www.readkong.com/page/2020-nsi-national-health-care-retention-rn-staffing-report-6269980

18. O'Brien-Pallas L, Murphy GT, Shamian J, Li X, Hayes LJ. Impact and determinants of nurse turnover: A pan-Canadian study. J Nurs Manag. 2010 Nov 1;18(8):1073–86.

19. He R, Liu J, Zhang WH, Zhu B, Zhang N, Mao Y. Turnover intention among primary health workers in China: A systematic review and meta-analysis. Vol. 10, BMJ Open. 2020. p. 37117.

20. Shen X, Jiang H, Xu H, Ye J, Lv C, Lu Z, et al. The global prevalence of turnover intention among general practitioners: a systematic review and meta-analysis. BMC Fam Pract. 2020;21(1):1–10.

21. Ito H, Eisen S V., Sederer LI, Yamada O, Tachimori H. Factors affecting psychiatric nurses' intention to leave their current job. Psychiatr Serv. 2001;52(2):232–4.

22. Al-Muallem N, Al-Surimi KM. Job satisfaction, work commitment and intention to leave among pharmacists: A cross-sectional study. BMJ Open. 2019 Sep 1;9(9).

23. Ayalew E, Workineh Y, Semachew A, Woldgiorgies T, Kerie S, Gedamu H, et al. Nurses' intention to leave their job in sub-Saharan Africa: A systematic review and meta-analysis. Heliyon. 2021 Jun 1;7(6).

24. Blaauw D, Ditlopo P, Maseko F, Chirwa M, Mwisongo A, Bidwell P, et al. Comparing the job satisfaction and intention to leave of different categories of health workers in Tanzania, Malawi, and South Africa. Glob Health Action. 2013;6:19287.

25. Ababa A. THE FEDERAL DEMOCRATIC REPUBLIC OF ETHIOPIA MINISTRY OF HEALTH NATIONAL HUMAN RESOURCE FOR HEALTH STRATEGIC PLAN FOR. 2016;

26. Nenko G, Vata PK. DEVELOPMENT RESEARCH Full Length Research Article ASSESSMENT OF HEALTH PROFESSIONALS ' INTENTION FOR TURNOVER AND DETERMINANT FACTORS IN YIRGALEM AND HAWASSA REFERRAL HOSPITALS , SOUTHERN ETHIOPIA Getachew Nenko and * Prabhanjan Kumar Vata. 2014;

27. Woldekiros AN, Getye E, Abdo ZA. Magnitude of job satisfaction and intention to leave their present job among nurses in selected federal hospitals in Addis Ababa, Ethiopia. PLoS ONE. 2022;17(6 June):1–12.

28. PRISMA [Internet]. [cited 2023 Jul 26]. Available from: http://www.prisma-statement.org/PRISMAStatement/

29. JBI Manual for Evidence Synthesis. JBI Manual for Evidence Synthesis. JBI; 2020.

30. Rayyan, Intelligent Systematic Review [Internet]. [cited 2022 Aug 15]. Available from: https://rayyan.ai/cite

31. Munn Z, MClinSc SM, Lisy K, Riitano D, Tufanaru C. Methodological guidance for systematic reviews of observational epidemiological studies reporting prevalence and cumulative incidence data. Int J Evid Based Healthc. 2015 Sep 1;13(3):147–53.

32. Project List | SRDR+ [Internet]. [cited 2023 Jul 27]. Available from: https://srdrplus.ahrq.gov/projects

33. van Rhee H, Suurmond R, Hak T. User Manual for Meta-Essentials: Workbooks for Meta-Analysis. SSRN Electron J. 2018 Feb 1;

34. Download - Meta-Essentials - Research Support - Erasmus Research Institute of Management - ERIM [Internet]. [cited 2022 Aug 26]. Available from: https://www.erim.eur.nl/research-support/meta-essentials/download/

35. MedCalc statistical software - free trial available [Internet]. [cited 2022 Aug 26]. Available from: https://www.medcalc.org/

36. de Cordova PB, Johansen ML, Grafova IB, Crincoli S, Prado J, Pogorzelska-Maziarz M. Burnout and intent to leave during COVID-19: A cross-sectional study of New Jersey hospital nurses. J Nurs Manag. 2022 Sep;30(6):1913–21.

37. He R, Liu J, Zhang WH, Zhu B, Zhang N, Mao Y. Turnover intention among primary health workers in China: a systematic review and meta-analysis. BMJ Open. 2020;10(10):e037117.

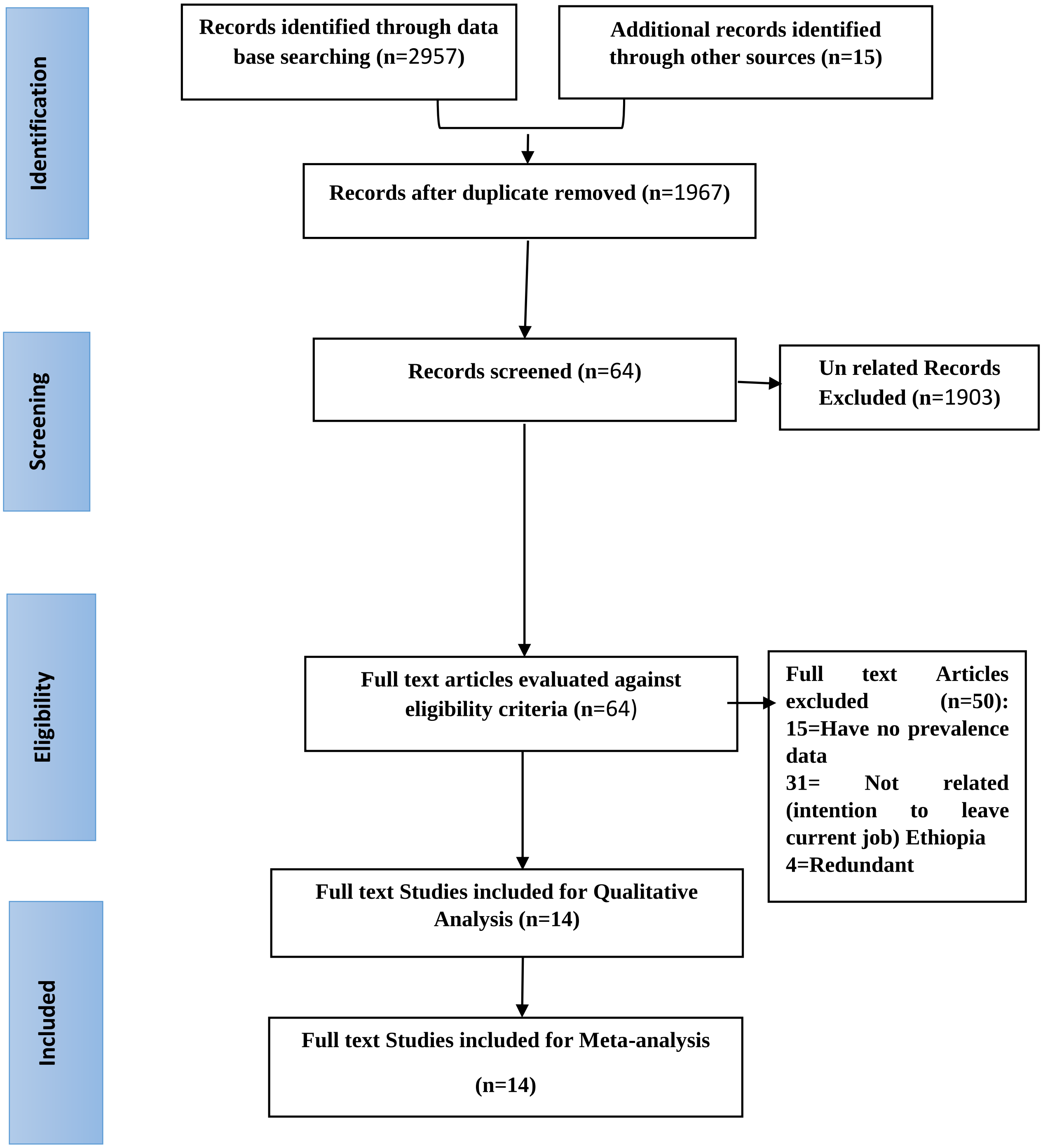

Fig 1. Flowchart presenting the study selection with the preferred reporting Items for systematic reviews and meta-analyses (PRISMA) guidelines on intention to leave among healthcare workers in Ethiopia, 2023.

Click here to access/download
Supporting Information
Annex 1.docx

Click here to access/download
Supporting Information
Annex 2.docx

Supporting Information

Click here to access/download
Supporting Information
PRISMA_2020_checklist-ITL.docx
