# Supplementary material for: Intention to leave the current health facility among healthcare workers in Ethiopia: Systematic review and meta-analysis
Source: PLOS Glob Public Health. 2024 Aug 14;4(8):e0003548. doi: 10.1371/journal.pgph.0003548 (PMC11324159; doi:10.1371/journal.pgph.0003548)
Supplement: Attachment — Submitted filename: Response to reviewers.docx [file pgph.0003548.s005.docx]

**Authors’ Response to Reviewers**

**Title:** Intention to Leave the Current Health Facility among Healthcare Workers in Ethiopia: Systematic Review and Meta-analysis

**Manuscript ID:** PGPH-D-23-02236

**Corresponding Author:** Gizew Dessie Asres ([gizew.dessie@gmail.com](mailto:gizew.dessie@gmail.com))

**Version 1**

**Date:** January 31, 2024

Dear Reviewers,

Thank you for your precious time you spent evaluating our manuscript to further improve the quality of the manuscript. We are very much grateful and have tried to respond accordingly. Manuscript preparation is an iterative process, in case you have got additional comments and suggestions, we are ready to correct as per your recommendations. The authors tried to address reviewers’ comments and suggestions point by point and indicated by track changes from the original manuscript, and also summarized response was given for general comments.

**Reviewer comment from Abstract**: I don’t understand this comment. How did enacting HR reforms impact universal health coverage? Please explain what is meant by universal health coverage in this context (background section, line 3).

**Authors Response for abstract comment:** Thank you for requesting details on how Enacting HR (Human Resources) reforms can have a significant impact on universal health coverage. Human resources in health refer to all individuals engaged in actions whose primary intent is to enhance health. These reforms involve policies and initiatives aimed at improving the recruitment, training, distribution, and retention of health workers, as well as their working conditions and performance.

**Achieving UHC requires three key components:**

***Coverage***: Ensuring that all individuals and communities have access to needed health services (including prevention, promotion, treatment, rehabilitation, and palliation) of sufficient quality to be effective while also ensuring that the use of these services does not expose the user to financial hardship.

***Quality:*** Health services should be effective, safe, people-centered, timely, equitable, and integrated.

***Financial protection***: Ensuring that the cost of using services does not put people at risk of financial hardship.

Here's how HR reforms can impact universal health coverage:

1. **Improved Access to Health Services**: By addressing shortages of health workers and ensuring they are deployed in areas where they are most needed, HR reforms can enhance access to health services, especially in underserved or remote areas.
2. **Enhanced Quality of Care**: Adequately trained and motivated health workers are essential for delivering high-quality health services. HR reforms that invest in training, continuing education, and professional development can improve the quality of care provided to patients.
3. **Increased Efficiency**: Streamlining administrative processes, optimizing staffing levels, and deploying health workers according to their skills and competencies can increase the efficiency of health service delivery, ensuring that resources are used effectively to meet the population's health needs.
4. **Reduced Financial Barriers**: Ensuring an adequate supply of health workers can help reduce waiting times for appointments and procedures, minimizing indirect costs associated with seeking care. Additionally, by providing services closer to communities, HR reforms can lower transportation costs for patients.
5. **Strengthened Health Systems**: HR reforms are integral to building resilient health systems capable of responding to the diverse health needs of populations. By investing in the health workforce, countries can strengthen the foundation of their health systems, making them better equipped to achieve universal health coverage.

In summary, enacting HR reforms can play a crucial role in advancing universal health coverage by improving access to services, enhancing quality of care, increasing efficiency, reducing financial barriers, and strengthening health systems overall.

**Reviewer comment from Abstract:** This statement is inaccurate. There is a plethora of research on intent to leave in healthcare. The authors cite several in the body of this manuscript (background section line 4).

**Authors Response for abstract comment:** Thank you for your important comment here. The word ‘limited’ is not necessary and removed based on suggestion. But the rest part of the statement is correct, there is fragmented or pocket research on intention to leave the current health facility.

**Reviewer comment from Abstract:** 1-2 significant figures after the decimal is sufficient (result section line 2).

**Authors Response for abstract comment:** Thank you for such line-by-line review of our manuscript. We accepted your comment and corrected it per your recommendation.

**Reviewer comment from Introduction:** You corrected the word ‘bourdon’ to ‘burden’ on paragraph 1 line 2.

**Authors Response for a comment from introduction:** That is very kind of you. We really appreciate your contribution to such extent.

**Reviewer comment from Introduction**: Requires reference (paragraph 1 line 5).

**Authors Response for a comment from introduction:** Thank you for your concern. But we have cited three references, please look at references 2-4 for this statement.

**Reviewer comment from Introduction**: Inaccurate sentence structure (paragraph 2 lines 1 to 2).

**Authors Response for a comment from introduction:** Thank you for this major comment and we corrected it by rewriting. Please see the track changes.

**Reviewer comment from Introduction**: How does a shortage of some disciplines create excess? (paragraph 2 lines 4 to 5)

**Authors Response for a comment from introduction:** We want to explain how actual turn over affects distribution of the already limited healthcare workers. When healthcare workers transfer from one facility to another, they may create shortage from the facility that released them and may be adequate or sometimes excess at the receiving facility. Here the word ‘excess’ is used to explain relative availability.

**Reviewer comment from Introduction:** Abbreviation not defined (Paragraph 6 line 6 ‘MoH’).

**Authors Response for a comment from introduction:** Thank you for the comment, we accepted and corrected it accordingly.

**Reviewer comment from Method:** please define all acronyms prior to first use (method section line 3 ‘JBI’).

**Authors Response for a comment from method:** Thank you for the point, we accepted and corrected it accordingly.

**Reviewer comment from inclusion and exclusion criteria:** Does this imply that studies examining healthcare workers leaving their job for another within the same facility were excluded? This needs to be clarified (exclusion criteria no. 4).

**Authors Response for a comment from exclusion criteria:** Thank you for raising this critical question. The objective of this study was to investigate intention to leave current working facility not the current job or position or profession within the facility. These are quite different but some scholars assessed together. So, these have to be excluded for this study.

**Reviewer comment from reference:** this is not an accurate citation (for references 25 and 26)

**Authors Response for a comment from reference:** Thank you for your comment, we corrected them accordingly.

**Reviewer 1 Comments to the Author**

Thank you for addressing the significant issue of intention to leave healthcare. I have two major concerns detailed below followed by minor suggestions.

• Can the authors elaborate on the factors associated with the intention to leave? Currently only a short paragraph is dedicated to this very important aspect of the manuscript and an underdeveloped section that would greatly improve the paper if more details were provided. What aspects of organizational policy were found to be significant? How was organizational policy defined across all studies? The term organizational policy is a very broad category and does not provide much information to the reader. Why were these factors the only factors assessed? Further can the authors break these results down by type of worker? Factors impacting nurses may be different from those working in the pharmacy or in food services. Also, Table 6 is missing

• The Discussion focuses primarily on repeating the results – there is very little explanation as to why the intent to leave is relatively high in Ethiopia and what could be done to address these issues. Further the authors only attribute differences with their study and others to study procedures, participants, and the study setting - what other reasons could be associated with intent to leave and how do they differ or are they similar with the current study? The Discussion section needs more development

• The paper overall is limited by inaccuracies and sentence structure, grammar, and wording.

• There are several statements highlighted in the attached that require accurate references.

• Some of the references are not accurately formatted

• Acronyms need to be defined prior to their first use

• Tables 2 is missing

Additional comments can be found in the attachment

**Author response to reviewer 1 comments:** Dear reviewer thank you for your line-by-line review and detailed comments to making the manuscript more enriched so that readers can get sufficient information. We made corrections per your recommendation and the changes are indicated by track changes from the original manuscript. Here is the point-by-point response to all raised queries by locating where these queries are addressed from the revised manuscript.

***Reviewer question 1***: Can the authors elaborate on the factors associated with the intention to leave? Currently only a short paragraph is dedicated to this very important aspect of the manuscript and an underdeveloped section that would greatly improve the paper if more details were provided. What aspects of organizational policy were found to be significant? How was organizational policy defined across all studies? The term organizational policy is a very broad category and does not provide much information to the reader. Why were these factors the only factors assessed? Further can the authors break these results down by type of worker? Factors impacting nurses may be different from those working in the pharmacy or in food services. Also, Table 6 is missing

***Authors response to reviewer’s question 1***: Thank you for this critical question. Based on accessed literatures, the authors identified possible factors for intention to leave their current facility as; autonomy, co- worker relationship, job satisfaction, organizational policy, payment and benefits system, performance appraisal, recognition, training and development opportunity and workload. But when statistically analyzed, only organizational policy was found to have significant relationship with the health workers intention to leave their current health facility. Organizational policy here considered as organizational justice which refers to the fairness of (resource) allocation within the organization. This encompasses the actions and decisions made by management, employees’ salary, opportunities for promotion and advancement, performance evaluation, etc. Employees expect the facility to be just, unbiased, and transparent about these aspects. If everything follows the proper course and the employees are treated fairly, they tend to be motivated and satisfied. Lack of organizational justice can demotivate employees and make them leave the organization. Those health workers who believe their facility has organizational justice tend to have lower intention to leave their current health facilities as indicated at table 6. Authors try to break the results by type of participant as indicated from table 5.

***Reviewer question 2:*** The Discussion focuses primarily on repeating the results – there is very little explanation as to why the intent to leave is relatively high in Ethiopia and what could be done to address these issues. Further the authors only attribute differences with their study and others to study procedures, participants, and the study setting - what other reasons could be associated with intent to leave and how do they differ or are they similar with the current study? The Discussion section needs more development

• The paper overall is limited by inaccuracies and sentence structure, grammar, and wording.

• There are several statements highlighted in the attached that require accurate references.

• Some of the references are not accurately formatted

• Acronyms need to be defined prior to their first use

• Tables 2 is missing

Additional comments can be found in the attachment

***Authors response to reviewer’s question 2:*** Thank you for your constructive and suggestive comments. All your comments are addressed on their respective sections with track changes on revised manuscript.

**Reviewer 2 Comments to the Author**

Dear Authors,

I read with great interest the manuscript received for evaluation. Your work investigates an important and current health issue. I have several comments aimed at enhancing its quality.

1. In Ethiopia, there is no representative and summarized data on health professionals’ intention to leave their current health facility. Some studies on intention to leave did not clearly state whether

it is the intention to leave their current profession or their current working facility. The paucity of

previous studies showed inconsistent prevalence that ranges from 30.4 % to 80.6% (26,27). Therefore, this systematic review and meta-analysis aimed to estimate the pooled prevalence of

health professionals’ intention to leave their current health facility and factors associated with it

in Ethiopia. But there is a systematic review and meta-analysis conducted in Ethiopia on this topic. What distinguishes this work from the existing literature/recently published systematic review and meta-analysis done in Ethiopia?

2. Your study conclusion needs more clarification.

3. The authors should revise the language to improve readability

**Author response to reviewer 2 comments:** We appreciate your concern about the similarity of the recently published systematic review on intention to leave. But our study is specifically on the intention to leave the current working health facility, which does not include intention to leave or change their profession within a health facility.

**Editor’s instruction and authors’ response to them**

Please include the following items when submitting your revised manuscript:

1. A rebuttal letter that responds to each point raised by the editor and reviewer(s). You should upload this letter as a separate file labeled 'Response to Reviewers'.

**Author Response to Editors suggestion:** Dear Editor thank you for your guiding suggestion on how to address reviewers’ comments. We did write a response to reviewers per your recommendation and the changes made were indicated by track changes at the original manuscript.

2. A marked-up copy of your manuscript that highlights changes made to the original version. You should upload this as a separate file labeled 'Revised Manuscript with Track Changes'.

**Author Response to Editors suggestion:** We did prepare the manuscript per your recommendation.

3. An unmarked version of your revised paper without tracked changes. You should upload this as a separate file labeled 'Manuscript'.

**Author Response to Editors suggestion:3** Thank you for your guiding suggestion and we did it according to your suggestion.
