# Supplementary material for: Intention to leave the current health facility among healthcare workers in Ethiopia: Systematic review and meta-analysis
Source: PLOS Glob Public Health. 2024 Aug 14;4(8):e0003548. doi: 10.1371/journal.pgph.0003548 (PMC11324159; doi:10.1371/journal.pgph.0003548)
Supplement: Attachment — Submitted filename: Response to reviewers.docx [file pgph.0003548.s006.docx]

**Authors’ Response to Reviewer 3**

**Title:** Intention to Leave the Current Health Facility among Healthcare Workers in Ethiopia: Systematic Review and Meta-analysis

**Manuscript ID:** PGPH-D-23-02236R1

**Corresponding Author:** Gizew Dessie Asres ([gizew.dessie@gmail.com](mailto:gizew.dessie@gmail.com))

**Version 2**

**Date:** June 4, 2024

Dear Reviewer 3,

Thank you for the precious time you spent evaluating our manuscript to further improve the quality of the manuscript. The authors tried to address your comments and suggestions point by point and indicated by track changes from the original manuscript.

**Reviewer 3 comments**

My comments and suggestions for the authors are as follows.

(1) Two important recent articles are not included in this study, at least I could not find any reference to them in the manuscript.

- Gebrekidan, A. Y. et al. (2023): https://bmjopen.bmj.com/content/bmjopen/13/5/e067266.full.pdf. In this one, the authors analysed a larger sample of articles, all about Ethiopia, all published after 2013.

- Zenebe, G. A. et al. (2024): https://www.ajol.info/index.php/emj/article/view/268493/253355. The main aim of this article is to examine health professionals’ intention to leave their jobs in public health facilities and its determinants in Ethiopia, using systematic review and meta-analysis.

The aim, structure, methods, and some of the results of the above articles are very similar to this study. For this reason, there is a risk that the novelty of the manuscript will be questioned. I therefore recommend that you include these two articles in the Introduction and Discussion of your paper, emphasizing the originality of your own work and its novelty compared to them.

(2) This study aims to assess the pooled prevalence of intention to leave their current health facility among health workers in Ethiopia. It is worth describing how pooled prevalence of intention to leave was calculated. For example, in the study of Zenebe, G. A. et al. (2024), this indicator “was calculated by dividing the total number of health professionals who intend to leave by the total sample size (number of health professionals included in the study) and multiplying by one hundred (100)”.

(3) During the study selection process, did you look for additional sources in the references of the articles already collected? I did not find any sign of this in the Methods section. If you did not use this ‘snowball’ technique, please explain why you did so.

(4) You wrote that you had collected articles and grey literature published between 2013 and 2022. Could you please explain why 2013 was chosen as the starting year?

(5) Some minor spelling and grammar issues should be revised (e.g. instead of “professional; autonomy”, did you want to write “professional autonomy”?).

**Authors’ Response to Reviewer 3**

**Response to Comment 1:** Dear reviewer 3, we really apricate your concern to effort duplication and nobility issues to our work. We have transparently passed all systematic review procedures, preliminary search at google scholar and PROSPERO to check similar works, there was no completed or started similar work there. Then we registered our protocol from PROSPERO with <https://www.crd.york.ac.uk/prospero/display_record.php?ID=CRD42022339438> .

The two recent articles are not exactly the same as our work. We have got ‘Gebrekidan, A. Y. et al. (2023): <https://bmjopen.bmj.com/content/bmjopen/13/5/e067266.full.pdf>’ during our search but we noticed that its finding did not address our research question why health workers want to change their working facility, might be the same level or different? It is not about why health workers want to change their profession. Actually, the above-mentioned review pooled intention to leave their health facility and profession together, which seems like adding oranges with apples. otherwise, it is about the intention to leave one’s job (might be from nursing to pharmacy or health officer or vis versa) but in our case intention to leave the working facility (might be from one health center to another health center or hospital or vis versa), we did not include those articles assessing intention to change their profession within the same facility or from one facility to another facility. Because factors related to changing one’s profession may not be addressed by health facilities but factors related to changing one’s facility can be addressed by individual health facilities. The second review, ‘Zenebe, G. A. et al. (2024): <https://www.ajol.info/index.php/emj/article/view/268493/253355> ‘ is also the same in content as the first one. However, we did not access it during our search as our last update was to 30 May 2023. Generally, these two review articles are not the same in their aim with our work.

**Response to comment 2**: Thank you for this important point, we addressed it in the manuscript with track changes in the introduction section.

**Response to comment 3**: Thank you for this important point, we addressed it in the methods part of the manuscript with track changes.

**Response to comment 4**: Thank you for such a detailed comment. We assumed that 10 years of evidence is enough to decide on the current intention to leave health facilities.

**Response to comment 5:** We really appreciate your time for line-by-line reading and detecting such a point, we did correct it per your suggestion.
